# Generating long-read sequences using Oxford Nanopore Technology from *Diospyros celebica* genomic DNA

**DOI:** 10.1186/s13104-021-05484-0

**Published:** 2021-02-27

**Authors:** Iskandar Zulkarnaen Siregar, Fifi Gus Dwiyanti, Rahadian Pratama, Deden Derajat Matra, Muhammad Majiidu

**Affiliations:** 1 Department of Silviculture, Faculty of Forestry and Environment, IPB University (Bogor Agricultural University), Bogor, Indonesia; 2 Molecular Science Research Group, Advanced Research Laboratory, IPB University (Bogor Agricultural University), Bogor, Indonesia; 3 Department of Biochemistry, Faculty of Mathematic and Natural Science, IPB University (Bogor Agricultural University), Bogor, Indonesia; 4 Department of Agronomy and Horticulture, Faculty of Agriculture, IPB University (Bogor Agricultural University), Bogor, Indonesia

**Keywords:** *Diospyros celebica*, Genome, ONT, Scaffolds, Long-read sequences

## Abstract

**Objectives:**

Development of sequencing technology has opened up vast opportunities for tree genomic research in the tropics. One of the aforesaid technologies named ONT (Oxford Nanopore Technology) has attracted researchers in undertaking testings and experiments due to its affordability and accessibility. To the best of our knowledge, there has been no published reports on the use of ONT for genomic analysis of Indonesian tree species. This progress is promising for further improvement in order to acquire more genomic data for research purposes. Therefore, the present study was carried out to determine the effectiveness of ONT in generating long-read DNA sequences using DNA isolated from leaves and wood cores of Macassar ebony (*Diospyros celebica* Bakh.).

**Data description:**

Long-read sequences data of leaves and wood cores of Macassar ebony were generated by using the MinION device and MinKnow v3.6.5 (ONT). The obtained data, as the first long-read sequence dataset for Macassar ebony*,* is of great importance to conserve the genetic diversity, understanding the molecular mechanism, and sustainable use of plant genetic resources for downstream applications.

## Objective

The third-generation sequencing from Oxford Nanopore Technologies (ONT) that is capable of generating long-read sequences was applied to fill the existing technological gaps, particularly with respect to capital cost, use of native DNA/RNA samples, simplicity, portability, ease of use for library preparation, etc. In particular, these technologies provided an on-site analysis that is of a significant advantage considering possible constraints due to existing gaps in the current regulation (e.g. Nagoya Protocol and others) on sample transfer permits either from field to laboratory, both within the country and overseas [1, 2]. The use of ONT on-site required fewer efforts in arranging the administration process for sample transfer, hence these functions enabled to accelerate data generation for various immediate needs, ones of which were urgent decision-making for species identification and conservation and even for on-site forensic investigation. The ONT could also be used in a hybrid system with other sequencing platforms, such as short-read sequencing in order to analyze missing fragments, structural variations, etc. [3]. In the tropics, research on the use of ONT to dissect biodiversity has been still limited due to the new finding scarcity, especially in regards to tree genomic variation analysis. Associated problems such as DNA/RNA yields and quality have been still consistently found depending on species and sample sources led by mainly more complex chemical compounds (such as phenols) and samples’ accessibility. In addition, site conditions might also influence the DNA yields forcing the use of only one general protocol across the samples. Macassar ebony—an endemic and vulnerable species in Sulawesi (Celebes), Indonesia, was utilized in the experiment and designed to determine the utilization efficacy aiming for long-read sequencing using samples from both leaves and small wood cores collected in Celebes [4]. Results of this study are presented in Table 1.

**Table 1:** Overview of data files/data sets

Label	Name of data file/data set	File types (extension)	Data repository and identifier (DOI or accession number)
Data file 1	List of ebony samples and Nanopore Native barcodes	Compressed XLSX file (.zip)	https://doi.org/10.6084/m9.figshare.13027991.v1 [13]
Data file 2	Raw reads statistics and plots (quality score, reads length, reads distribution)	Compressed TXT, PNG and HTML files (.zip)	https://doi.org/10.6084/m9.figshare.13028069.v1 [14]
Data file 3	Polished De novo genome contig assemblies of both assembler	Compressed FASTA files (.zip)	https://doi.org/10.6084/m9.figshare.13031195.v1 [15]
Data file 4	Statistics of corrected contig assemblies with *Diospyros celebica* chloroplast	Compressed PDF file (.zip)	https://doi.org/10.6084/m9.figshare.13028177.v1 [16]
Data file 5	Statistics of corrected contig assemblies with *Diospyros lotus* genome	Compressed PDF file (.zip)	https://doi.org/10.6084/m9.figshare.13028180.v1 [17]
Data file 6	Constructed scaffolds from both assembler	Compressed FASTA files (.zip)	https://doi.org/10.6084/m9.figshare.13031702.v1 [18]
Data file 7	Statistics of scaffolds assemblies with *Diospyros celebica* chloroplast	Compressed PDF file (.zip)	https://doi.org/10.6084/m9.figshare.13031693.v1 [19]
Data file 8	GenBank annotation from scaffolds assemblies	Compressed GB files (.zip)	https://doi.org/10.6084/m9.figshare.13031708.v1 [20]
Data file 9	Annotation visualization from scaffolds assemblies	Compressed JPG files (.zip)	https://doi.org/10.6084/m9.figshare.13031714.v1 [21]
Data set 1	Raw genomic DNA reads (trimmed barcode)	FASTQ files (.fastq)	DNA Data Base of Japan (DRP006615) https://identifiers.org/insdc.sra:DR006615 [22]

## Data description

Total genomic DNA from 15 individuals of Macassar ebony (*Diospyros celebica* Bakh.) leaves (n = 11) and wood core (n = 4) collected by using Pickering Punch in three provinces in Indonesia, namely Central Sulawesi, West Sulawesi, and South Sulawesi, were extracted using a modified CTAB methods [5] in which the CTAB buffer contained CTAB 10%, Tris HCl, NaCl 5 M, EDTA 0.5 M, PVP 1%, β-Mercaptoethanol, and dH_2_O. DNA quality was evaluated by electrophoresis using a Gel Doc EZ System (Bio-Rad, USA) and DNA concentration was measured by using NanoPhotometer NP80 (IMPLEN, Germany).

The library preparation of genomic DNA sample was followed the Nanopore Protocol for Native barcoding genomic DNA (with EXP-NBD104 and SQK-LSK109), version NBE_9065_v109_revJ_23May2018. Sequencing was done in two rounds using two flowcells (FLO-MIN106). The list of samples per flowcell, as well as the native barcode (NBD01–NBD12) used in the study, were listed in Data File 1.

The sequencing run of genomic DNA samples was performed using the MinION device and MinKnow v3.6.5. Sequencing was terminated after no more pores actively sequenced the DNA. The high-accuracy base-calling mode was used to base-call the signal in FAST5 files and outputted FASTQ files. Samples were separated according to each barcode, where afterwards the barcodes were set to automatically trimmed from the reads (Data set 1). All samples were combined using *cat* command on Linux Mint terminal and analyzed by using NanoStat v1.2.1 to assess the reads quality and reads' statistics. Meanwhile, distribution plots were generated by using NanoPlot v1.31.0 [6] (Data file 2). We obtained 302 567 reads with 99.5% reads quality > Q7 (nanopore default passed quality). After statistic inspection, all reads quality was filtered through NanoFilt v2.7.1 [6]. Reads with Q-score lower than 7 and less than 500 bp were filtered out, with parameter *–headcrop* and *–tailcrop* of 10 were applied. Reads filtering resulted in 134 220 reads, then subject to correction, trimming and De novo assembly using Canu v2.0 [7] with option of *genome Size* = *800 m.* Another De novo long-reads assembler was applied to compare the contig assemblies from plant DNA using SMARTdenovo [8] with minimum read length (*−J*) 2 000*.* SMARTdenovo utilized corrected reads 'step from Canu correction stage, thus expected to result in better outcome than the Canu assembly's. The contig assemblies were 358 (N50 6.5 kb, GC 39.91%) and 39 (N50 12.7 kb, GC 41.14%) for Canu and SMARTdenovo respectively. The draft assembly then was polished (corrected) against the individual sequencing reads using medaka_consensus v1.0.3 [9] with parameter model for nanopore sequencing (*−m*) *r941_min_high_g330* (Data file 3). The resulting polished assembly statistics was calculated using QUAST v5.0.2 [10], with references of *Diospyros celebica* chloroplast (Data file 4) and *Diospyros lotus* genome (Data file 5). This statistic calculation informed how much reference genome fraction covered by the contig assemblies. The polished contigs then were chosen to construct scaffold using LINKS v1.8.7 [11] with default parameter (Data file 6). The resulted scaffolds were 266 (N50 11.3 kb, GC 39.91%) and 33 (N50 17.8 kb, GC 41.14%) for Canu and SMARTdenovo respectively. The longest scaffolds assemblies from both assembler (Canu 141.6 kb, SMARTdenovo 145 kb) were validated with QUAST v5.0.2 with *D. celebica* chloroplast to check the genome fraction that scaffolds covered (Data file 7). These scaffolds assemblies were then annotated by using GeSeq platform for Organellar Genomes [12], resulted in the GenBank annotation (Data file 8) and their visualization (Data file 9).

## Limitations

The long-read sequencing of the Macassar ebony tree equipped with nanopore sequencing was quite challenging. Extraction of genomic DNA shall be optimized to obtain high-quality gDNA without excessive fragmentation. The resulting fragmented DNA required to be removed prior to library preparation as they might occupy nanopores within the flowcells and cause too many short reads across the sequencing outputs. The library preparation shall be optimized as well, for example, the DNA concentration was measured with a spectrophotometer, which could lead to a biased number of the aforementioned concentration. DNA fluorometer was preferred to accurately calculate DNA concentration. The correct DNA concentration loaded into the MinION flowcell would enable the optimal DNA sequencing process and pores occupancy. Achieving higher sequencing throughput is necessary to improve the read accuracy limitation of MinION as been observed in this study.

## Data Availability

The data described in this Data note can be freely and openly accessed on figshare [9, 10] (https://doi.org/10.6084/m9.figshare.13027991.v1, https://doi.org/10.6084/m9.figshare.13028069.v1, https://doi.org/10.6084/m9.figshare.13031195.v1, https://doi.org/10.6084/m9.figshare.13028177.v1, https://doi.org/10.6084/m9.figshare.13028180.v1, https://doi.org/10.6084/m9.figshare.13031702.v1, https://doi.org/10.6084/m9.figshare.13031693.v1, https://doi.org/10.6084/m9.figshare.13031708.v1, https://doi.org/10.6084/m9.figshare.13031714.v1, https://identifiers.org/insdc.sra:DRP006615). Please revert to Table 1 and references list [11–20] for details and links to the data.

## Abbreviations

ONT: Oxford Nanopore Technology
DNA: Deoxyribonucleic acid
RNA: Ribonucleic acid
CTAB: Cetyl Trimethyl Ammonium Bromide
HCl: Hydrochloric acid
NaCl: Sodium chloride
EDTA: Ethylenediaminetetraacetic acid
PVP: Polyvinylpyrrolidone
gDNA: Genomic deoxyribonucleic acid
